# Development of a general methodology for labelling peptide–morpholino oligonucleotide conjugates using alkyne–azide click chemistry†
†Electronic supplementary information (ESI) available: Experimental section and supplementary figures. See DOI: 10.1039/c3cc46067c



**DOI:** 10.1039/c3cc46067c

**Published:** 2013-09-25

**Authors:** Fazel Shabanpoor, Michael J. Gait

**Affiliations:** a MRC Laboratory of Molecular Biology , Cambridge , CB2 0QH , UK . Email: mgait@mrc-lmb.cam.ac.uk

## Abstract

We describe a general methodology for fluorescent labelling of peptide conjugates of phosphorodiamidate morpholino oligonucleotides (PMOs) by alkyne functionalization of peptides, subsequent conjugation to PMOs and labelling with a fluorescent compound (Cy5-azide). Two peptide–PMO (PPMO) examples are shown. No detrimental effect of such labelled PMOs was seen in a biological assay.

## Abstract 2

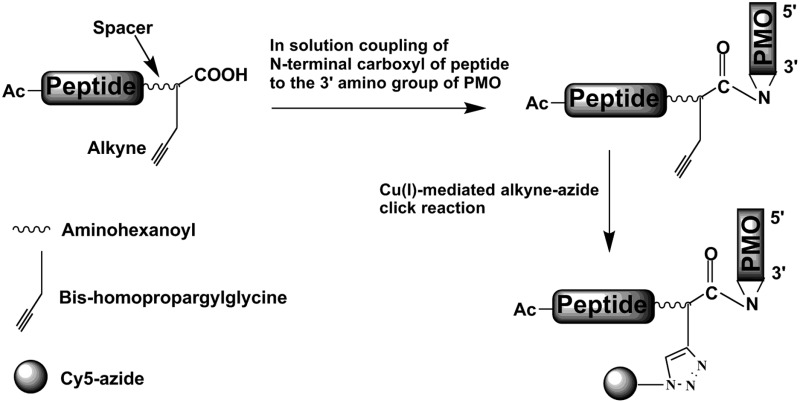
A general and convenient solid-phase synthesis method using Cu(i)-mediated alkyne–azide click chemistry for attachment of an azide derivative of a fluorescent label to an alkyne derivative of a peptide conjugated to a morpholino oligonucleotide (PMO).

Phosphorodiamidate morpholino oligonucleotides (PMOs) (Fig. 1) are synthetic oligonucleotide analogues that contain a charge-neutral backbone^1^ and which have gained great attention because of their steric blocking antisense properties. They are very attractive for use in drug development because of their low toxicity and high enzymatic stability.^2^ For example, they have been used to manipulate gene expression by steric blocking of mature mRNA translation^3^ or by altering pre-mRNA splicing.^4^ PMOs have been used in the development of various antisense therapies, including treatment of neuromuscular diseases^5,6^ and viral and bacterial infections.^7,8^ In the case of Duchenne muscular dystrophy (DMD), PMOs have shown excellent ability to induce exon-skipping of pre-mRNA both in cell cultures^5^ and in animal models^9–11^ and a 30 mer PMO has been in clinical trials in the UK and USA.^12,13^ PMOs have also been used for exon inclusion in a mouse model of spinal muscular atrophy.^14^ In order to enhance cellular and *in vivo* delivery of PMOs, much recent work has focused on the use of cell-penetrating peptides (CPPs), particularly those that are Arg-rich.^15,16^ Their use results in significantly improved cellular uptake^17^ as well as much improved *in vivo* properties.^18,19^ CPPs are covalently conjugated to 5′-amino-functionalized PMO or to the 3′-secondary amine of the PMO.^18–20^

**Fig. 1:**
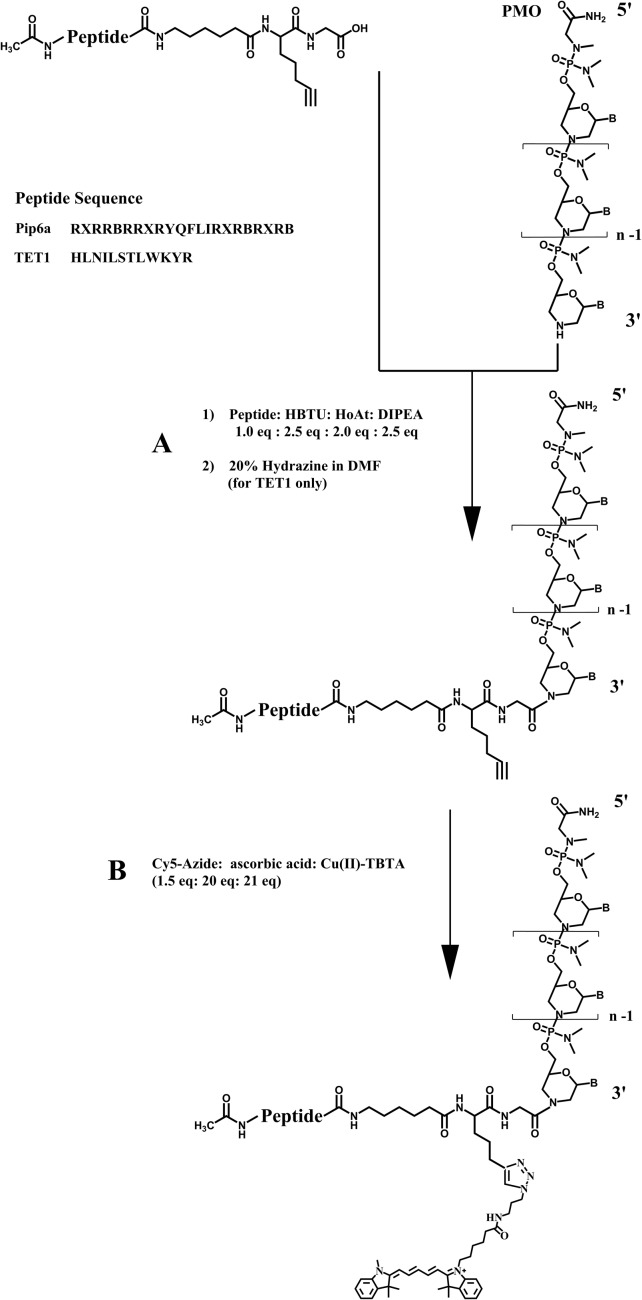
Activation of the C-terminal carboxyl group of TET1 and Pip6a peptides using HBTU–HOAt and coupling to the 3′-secondary amine of the PMO (A.1). A spacer (aminohexanoic acid) was inserted between the alkyne residue and the peptide sequence. In the case of TET1, the ivDde protecting group of lysine was removed by addition of 20% hydrazine to the reaction mixture (A.2). The labelling was carried out by clicking the Cy5-azide (1.5 eq.) to the alkyne side-chain using Cu(i) (21 eq.) mediated click chemistry in the presence of sodium ascorbate (20 eq.) (B).

The detection of PMOs in tissues after *in vivo* administration of PPMOs is currently based on laborious HPLC isolation of PMOs followed by a hybridization detection assay.^21^ No *in vivo* imaging or a biodistribution method for PMOs or PPMOs using fluorescence has been reported to date. Furthermore, fluorescence imaging in cells has been restricted to the use of the 3′-fluorescein, 5′-peptide functionalized PMO,^22^ since PMO synthesis has only been possible hitherto commercially and the only fluorescently labelled PMO available contains a 3′-fluorescein, which is poor for cellular visualization due to the pH sensitivity of its fluorescence. Such a label is also unsuitable for animal imaging, since near/far infrared fluorescent labels are required to eliminate autofluorescence from cells or tissues. Therefore, we sought to develop a general methodology for labelling PPMOs that utilizes commercially available, unfunctionalized PMOs and which would retain the label close to the PMO in the event of peptide proteolysis *in vivo* or intracellularly. Our novel method involves PMO conjugation with a synthetic peptide containing an appropriate C-terminal alkyne derivative, which is subsequently reacted by copper-mediated alkyne–azide click chemistry.^23,24^

Two model peptides (Pip6a and TET1) were chosen for conjugation to PMOs and subsequent labelling with a fluorescent (Cy5-azide) dye, which is suitable both for cells^25^ and *in vivo* imaging.^26^ In the first step, peptides were synthesized as C-terminal acids, purified by RP-HPLC and obtained in good yields (60% and 55% respectively). In each case, the C-terminal residue was Gly followed by a commercially available bis-homopropargylglycine derivative that contained the alkyne functionality. An aminohexanoyl spacer was also introduced after alkyne functionalization before continuation of the solid phase assembly of the model peptide. In the case of the TET1 peptide, the Lys residue was protected by ivDde.

Conjugation of the peptides to the PMO was carried out in solution (Fig. 1A). The Pip6a-(alkyne)-PMO was purified on a cation-exchange column using a phosphate-based buffer with sodium chloride as an eluent. The TET1-(alkyne)-PMO conjugate required initial hydrazine treatment to remove the ivDde group and was then purified by RP-HPLC using a TFA-based buffer. Any trifluoroacetate counterions were removed by addition of 5 mM HCl followed by membrane filtration to remove dissociated trifluoroacetate ions and excess HCl. Note that lyophilisation of TET1-(alkyne)-PMO from this solution (instead of filtration) led to complete degradation of the PMO part of the PPMO and was thus avoided (ESI,† Fig. S1). The final conjugates were obtained in yields of 35% for Pip6a-(alkyne)-PMO and 46% for TET1-(alkyne)-PMO.

The Cy5-azide fluorescent dye was coupled to the alkyne functionality for both PPMOs. Each conjugation reaction was completed in about 2 h and the conversion efficiency of the labelling was found to be essentially quantitative as judged by the disappearance of unlabelled PPMO by analytical RP-HPLC. The overall isolated yields following RP-HPLC were determined by measuring the UV absorbance at 265 nm and found to be 63% and 51% for Pip6a-(Cy5)-PMO and TET1-(Cy5)-PMO respectively. In each case, the labelled PPMO eluted about 3 min later than the unlabelled PPMO on the RP-HPLC column (for example Pip6a-(Cy5)-PMO, Fig. 2A). The labelled conjugates were characterised using MALDI-TOF mass spectroscopy (Fig. 2B and C).

**Fig. 2:**
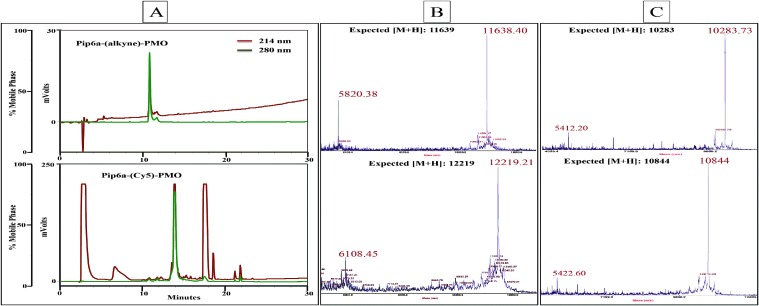
RP-HPLC chromatogram of Pip6a–PMO bearing an alkyne moiety (A top trace) which has been labelled with Cy5-azide using copper(i) mediated click chemistry (A bottom trace). The gradient used was 10–90% buffer B (acetonitrile–water containing 0.1% trifluoroacetic acid (9 : 1)) over 30 min at a flow rate of 1.5 ml min^–1^. MALDI-TOF mass spectroscopy profile of unlabelled and Cy5-labelled Pip6a–PMO (B) and TET1-PMO (C).

In order to determine the effect of incorporation of the alkyne moiety and conjugation of the bulky dye on the biological activity of the PPMOs, they were tested for the efficacy in an exon-skipping assay using the H2K *mdx* mouse muscle cell line.^18^ The levels of exon-23 skipping of the dystrophin gene were measured for peptide–PMO, peptide-(alkyne)-PMO and peptide-(Cy5)-PMO and each had a similar level of exon-skipping activity (Fig. 3A and B). This showed that neither the introduction of the alkyne moiety nor of the Cy5-label between the peptide and PMO separated by an aminohexanoic acid spacer has any impact on the internalization activity of the peptide and the exon-skipping efficacy of the PMO.

**Fig. 3:**
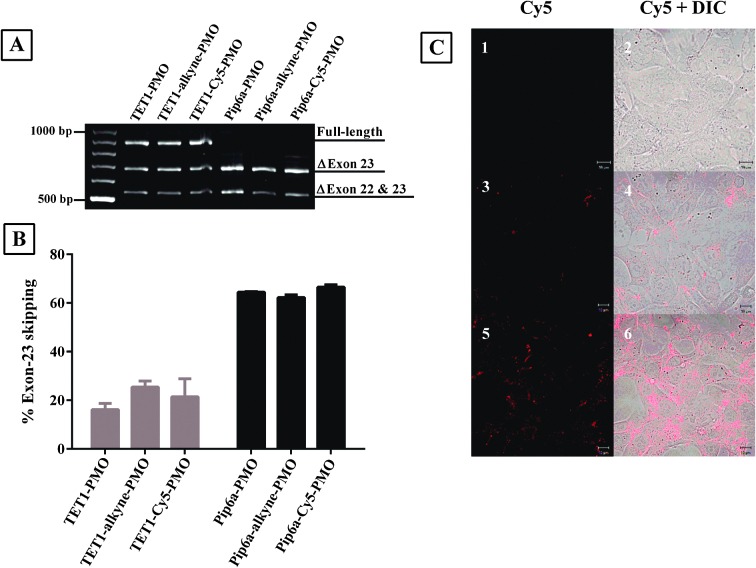
Representative products of nested RT-PCR analysis of dystrophin exon-skipping in H2K mdx cells treated with peptide–PMO, peptide-(alkyne)-PMO and peptide-(Cy5)-PMO at 1 μM for 4 h (A). Densitometric analysis of nested RT-PCR products for percentage of dystrophin exon-23 skipping (B). Confocal microscopy images of untreated (C-1,2), TET1-(Cy5)-PMO (C-3,4) and Pip6a-(Cy5)-PMO treated mouse brain endothelial cells (bEND5) (C-5,6). Left panel: Cy5 and right panel: Cy5 and DIC overlay.

Confocal laser scanning microscopy was used to estimate the cellular uptake of Cy5-labelled peptide–PMO conjugates in mouse brain endothelial cells (bEND5). Pip6a-(Cy5)-PMO showed the highest level of fluorescence (Fig. 3C5,6) which was lower for TET1-(Cy5)-PMO (Fig. 3C3,4). The level of fluorescence intensity of each conjugate was shown to correlate with their respective exon-skipping activities. This also shows that incorporation of a bulky dye between the peptide and PMO does not have an impact on the cellular uptake of the peptide–PMO conjugate.

In this study, we have used alkyne–azide click chemistry to develop a method for fluorescent labelling of PPMOs and exemplified this using a Cy5 fluorophore. The method is applicable in principle to any other fluorescent label in which an azide substituent is available to react with the PPMO *via* an alkyne moiety. Cy5-labelled PPMO now allows the study of *in vivo* biodistribution in mice, hitherto not possible. Incorporation of an alkyne functional group provides great flexibility also for conjugation of a PPMO with moieties such as biotins, lipids, or other agents that may alter *in vivo* properties, as well as additional cell targeting peptides or even for attachment of a second PMO to a different target. Studies of these types are in progress.

MJG acknowledges that this work was supported by the Medical Research Council (MRC programme no. U105178803). FS is a recipient of a C. J. Martin Fellowship from the Australian National Health and Medical Research Council. We thank Matthew Wood and colleagues (University of Oxford) for collaborations that inspired this work.
