# Supplementary material for: Development of a general methodology for labelling peptide–morpholino oligonucleotide conjugates using alkyne–azide click chemistry
Source: Chem Commun (Camb). 2013 Sep 25;87(49):10260–2. doi: 10.1039/c3cc46067c (PMC3816334; doi:10.1039/c3cc46067c)
Supplement: Supplementary file 1 [file CC-049-C3CC47264G-s001.pdf]

This journal is © The Royal Society of Chemistry 2013

SUPPORTING INFORMATION

Development of a general methodology for labelling peptide conjugates of morpholino (PMO) oligonucleotides using alkyne-azide click chemistry

Fazel Shabanpoor and Michael J. Gait

Medical Research Council, Laboratory of Molecular Biology, Francis Crick Avenue, Cambridge, CB2 0QH

General chemical methods

9-Fluroenylmethoxycarbonyl (Fmoc) protected L-α-amino acids, Fmoc-Lys(ivDde)-OH, Fmoc-6-aminohexanoic acid, benzotriazole-1-yl-oxy-tris-pyrrolidino-phosphonium hexafluorophosphate (PyBOP), 2-(1H-benzotriazol-1-yl)-1,1,3,3-tetramethyluronium hexafluorophosphate (HBTU), Fmoc-Gly-Wang resin (100-200, 0.79 mmol g^{-1}) were obtained from Merck (Hohenbrunn, Germany). Fmoc-L-bis-homopropargylglycine-OH was purchased from Chiralix (Nijmegen, Germany). HPLC grade acetonitrile, methanol and synthesis grade *N*-methyl-2-pyrrolidone (NMP) were from Fisher Scientific (Loughborough, UK). Peptide synthesis grade *N, N*-dimethylformamide (DMF) and diethyl ether were obtained from VWR (Leicestershire, UK). Piperidine and trifluoroacetic acid (TFA) were obtained from Alfa Aesar (Heysham, England). 3, 6-dioxa-1,8-octanedithiol (DODT), triisopropylsilane (TIPS), diisopropylethylamine (DIPEA), acetic anhydride, tris[(1-benzyl-1H-1,2,3-triazol-4-yl)methyl]amine (TBTA), sodium-L-ascorbate and copper (II) sulphate pentahydrate, sinapinic acid and α-cyano-4-hydroxy-cinnamic acid were from Sigma-Aldrich (St. Louis, MO, USA). Chicken Embryo Extract and horse serum were from Sera Laboratories International Ltd (West Sussex, UK). γ-Interferon was obtained from Roche Applied Science (Penzberg, Germany).

Peptide synthesis

Two peptide sequences (Pip6a [1]: RXRRBRRXRYQFLIRXRBRXRB (B: β-alanine, X: aminohexanoic acid) and Tet1[2]: HLNILSTLWKYR) were synthesized as C-terminal acids on an Fmoc-Gly-Wang resin (100-200, 0.79 mmol/g) using Fmoc chemistry [3]. The first amino acid, Fmoc-L-bis-homopropargylglycine-OH, was coupled manually using a 1.5 molar excess following HBTU activation. The

remainder of each sequence was assembled on a CEM Liberty^{TM} microwave peptide synthesizer (Buckingham, UK). The side chain-protecting groups used were trifluoroacetic acid-labile, except for 1-(4,4-dimethyl-2,6-dioxocyclohex-1-ylidene)-3-methylbutyl (ivDde) for Lys which was hydrazine-labile. Peptides were synthesized on a 0.1 mmol scale using a 5-fold excess of Fmoc-protected amino acids (0.5 mmol), which were activated using PyBOP (5-fold excess) in the presence of DIEA. Piperidine (20% v/v) in DMF was used to remove Nα-Fmoc protecting groups. The coupling was carried out once at 75 °C for 5 min, except for histidine which was coupled at 50 °C for 10 min at 60 watt microwave power. The deprotection was carried out at 75 °C twice, once for 30 sec and then for 3 min at 35 watt microwave power. The N-terminus of the solid phase-bound peptide was acetylated with acetic anhydride. The absence of a red colouration of the resin in the 2,4,6-trinitrobenzenesulphonic acid (TNBSA) test [4] indicated the success of N-terminus capping. The resin-bound peptide was cleaved from the solid support by treatment with a cocktail of trifluoroacetic acid (TFA): 3, 6-dioxa-1, 8-octanedithiol (DODT): H_2O: triisopropylsilane (TIPS) (94%:2.5%:2.5%:1%), 10 ml) for 2 h at room temperature. Excess TFA was evaporated off by blowing nitrogen into the peptide-TFA solution. The cleaved peptide was precipitated by addition of ice-cold diethyl ether and centrifuged at 3000 rpm for 2 min. The peptide pellet was washed in ice-cold diethyl ether at least three times. Crude peptides were dissolved in water, analyzed and purified by RP-HPLC on Phenomenex Jupiter columns (4.6 × 250 mm, C18, 5μm) and (21.2 × 250 mm, C18, 10μm) respectively. 0.1% trifluoroacetic acid in water was used as solvent A and acetonitrile / water containing 0.1% trifluoroacetic acid (9:1) as solvent B. A gradient of 10 to 70 % B over 30 min was used at a flow rate of 1.5 ml min^{-1} for the analytical and 10 ml min^{-1} for the preparative column. Both peptides were purified to greater than 90% purity as determined by analytical RP-HPLC.

Peptide-PMO conjugation and purification

A 25-mer PMO antisense sequence for mouse dystrophin exon-23 (GGCCAAACCTCGGCTTACCTGAAAT) was purchased from Gene Tools Inc. (Philomath, USA). Peptides were conjugated to the 3'-end of the PMO through its C-terminal carboxyl group. This was activated using a 2.5 and 2-fold excess of HBTU and HOAt in NMP respectively in the presence of 2.5 eq. of DIEA and a 2-fold

excess of peptide over PMO dissolved in dimethylsulfoxide (DMSO) was used. The reaction was carried out at 40 °C for 2 h. The ivDde protecting group of Lys in Tet1 was removed by addition of 20 eq. of hydrazine (cf ivDde) to the reaction mixture and incubation at room temperature for 30 min. The reaction was stopped by addition of water. Pip6a-(alkyne)-PMO was purified on a high-resolution (HR)-16 cation-exchange column (GE Healthcare, USA) using 25 mM sodium phosphate buffer (pH 7.2) containing 25% acetonitrile. 1 M NaCl solution was used to elute the peptide from the column at a flow rate of 6 ml min^{-1}. Due to the lack of sufficient charge to retain Tet1-(alkyne)-PMO conjugate on an ion-exchange column, it was purified using RP-HPLC. In the case of Pip6a-(alkyne)-PMO, the excess salts were removed by filtering the fractions collected after ion-exchange using an Amicon® Ultra-15 3K centrifugal filter device. Removal of any TFA counter-ions from the Tet1-(alkyne)-PMO conjugate was carried out by dissolving the lyophilized conjugate in 5 mM HCl solution followed by filtration. The concentrate (~250 µl) was further diluted by addition of 10 ml of 5 mM HCl solution and filtered again. Finally, the concentrate was washed twice with de-ionised water before lyophilisation.

Post-conjugation labelling of Peptide-PMO conjugates

The alkyne functionalized PPMOs were labelled with Cy5-azide (Lumiprobe Life Science Solutions, Florida, USA) using copper (I) mediated alkyne-azide click chemistry. PPMOs were dissolved in 30% DMSO followed by addition of Cy5-azide (1.5 eq. in DMSO). Sodium ascorbate (20 eq. as a 20 mM solution) was added and the reaction mixture was vortexed thoroughly followed by addition of copper (II)-TBTA (21 eq. as a 20 mM solution) which was prepared by mixing equal amounts of 40 mM $CuSO_4.5H_2O$ dissolved in water with 40 mM TBTA ligand dissolved in DMSO. The final volume of DMSO in the mixture was kept below 50%. The vial was flushed with nitrogen gas and left at room temperature for 2 h. The progression of the reaction was monitored by MALDI-TOF mass spectrometry. After the completion of the reaction, the Cy5-labelled PPMOs were purified and desalted as described above.

MALDI-TOF mass spectral characterization of peptides and PPMO

Matrix-assisted laser desorption ionization time-of-flight mass spectrometry (MALDI-TOF MS, ABI Voyager DE Pro) was used to characterize the peptides and PPMOs. Mass accuracy on this equipment is expected to be ± 0.1%. Based on the

molecular masses of the peptides and PPMOs, α-cyano-4-hydroxy-cinnamic acid matrix was used to analyse the peptides and PPMOs were analysed with sinapinic acid. The matrices were made up in 70% acetonitrile containing 0.05 % TFA. The concentration of the PPMOs was determined by measuring the molar absorption of the conjugates at 265 nm in 0.1 N HCl solution.

Cell culture and transfection

Mouse H2K/mdx myoblasts were maintained in high-glucose Dulbecco's modified Eagles's medium (DEME) supplemented with 20% foetal calf serum, 2% chicken embryo extract (CEE) and 0.002% of interferon-γ at 33 °C. The myoblast cells were differentiated into myotubes for the exon-skipping assay. Myoblasts were plated at a density of 5×10^5 cells per 1 ml per well in 24 well plates that were pre-coated with 0.01% gelatin. Cells were allowed to reach >90% confluent in DEME with 20% FCS / 2% CEE / 0.002 % interferon-γ at 33 °C. They were washed with PBS twice and the media was changed to DEME supplemented with 5% horse serum (HS) and transferred to 37 °C. Cells were washed with PBS and fresh 5% HS media was added daily for 3-4 days until a homogeneous layer of myotubes was formed. The PPMO concentrations were made up in serum-free Opti-MEM and 350 µl was added to each well as duplicates and incubated for 4 hr at 37 °C. The transfection medium was then replaced with DMEM / 5% horse serum and cells incubated for a further 20 h at 37 °C. Cells were washed with PBS once and 0.5 ml of TRI RNA isolation reagent was added to each well. Cells were frozen at -80 °C for 1 h.

For fluorescent uptake studies, mouse brain endothelial cells (bEND5) were maintained in DMEM / 10% FCS and seeded at a density of 25,000 cell/well of an 8 wells lab-tek chamber slide precoated with poly-D-lysine. The cells reached 80% confluency after 24 hr. They were treated with Cy5-labelled conjugates made up at a concentration of 1 µM in MEM without phenol red and serum for 1 hr at 37 °C. Cells were washed with PBS three times. The fluorescent images of cells were taken using a Zeiss LSM780 confocal laser scanning microscope (Cambridge, UK) with plan-apochromat 63×/1.4 oil objective. The images were acquired at the excitation of 633 nm and emission of 638-679 nm.

RNA extraction and nested RT-PCR analysis

This journal is © The Royal Society of Chemistry 2013

Total cellular RNA was extracted using TRI reagent with an extra further precipitation with ethanol. The purified RNA (500 ng) was used as a template for RT-PCR by use of a OneStep RT-PCR Kit (Roche, Indianapolis, USA). The primer sequences used were Exon20F0 5'-CAGAATTCTGCCAATTGCTGAG-3' and Exon26Ro 5'- TTCTTCAGCTTGTGTCATCC-3'. The cycle conditions for the initial reverse transcription were 50°C for 30 min and 94°C for 7 min for 1 cycle followed by 30 cycles of 94°C for 20 sec, 55°C for 40 sec and 68°C for 80 sec. The cDNA (1 μl) from the first RT-PCR step was used as the template for the second PCR step. The primer sequences were Exon20Fi 5'-CCCAGTCTACCACCCTATCAGAGC-3' and Exon2Ri 5'-CCTGCCTTTAAGGCTTCCTT-3'. The amplification was carried out using 0.5 U of SuperTAQ in 25 cycles at 94°C for 30 sec, 55°C for 1 min and 72°C for 1 min. The products were separated by electrophoresis using 1.5% agarose gel.

Data analysis

The images of agarose gels were taken on a Molecular Imager ChemiDoc™ XRS^{+} imaging system (BioRad, UK) and the images were analysed using Image Lab (V4.1). Graphpad PRISM 6 was used to analyze and plot the exon-skipping assay data, which were expressed as percentages of exon-23 skipping from at least three independent experiments. The confocal microscopy images were processed with ZEN2010 software.

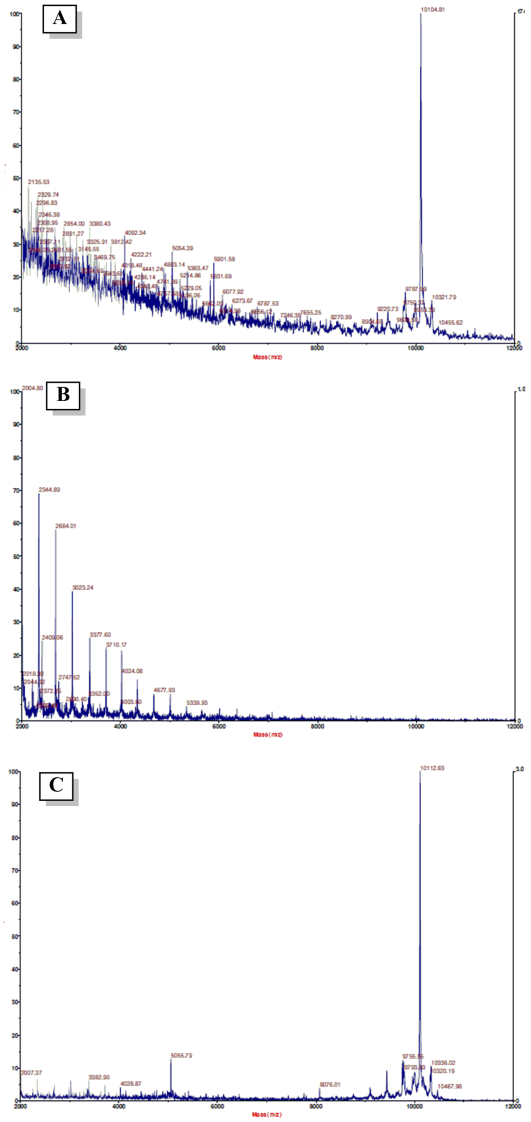

Supp.Fig. 1 MALDI-TOF mass spectra of TET1-PMO conjugate. (A) Mass spectrum profile of TET1-PMO conjugate after purification using 0.1% TFA-based buffer. (B) After lyophilisation, the conjugate was dissolved in 5 mM HCl and lyophilised again, which led to the degradation of the PMO. (C) The lyophilised TET1-PMO was dissolved in 5 mM HCl and desalted using a 3K Amicon filtration device. The concentrate was further diluted in 5 mM HCl and filtered again. The concentrate was washed twice with H_2O by adding deionised H_2O and filtered. The concentrated was lyophilised and the conjugate was found to be intact.

References

1. Betts, C., et al., *Pip6-PMO, A New Generation of Peptide-oligonucleotide Conjugates With Improved Cardiac Exon Skipping Activity for DMD Treatment.* Mol. Ther. Nucl. Acids, 2012. 1: p. e38.
2. Liu, J.K., et al., *A novel peptide defined through phage display for therapeutic protein and vector neuronal targeting.* Neurobiology of Disease, 2005. 19(3): p. 407-418.
3. Amblard, M., et al., *Methods and protocols of modern solid phase peptide synthesis.* Mol. Biotechnol., 2006. 33(3): p. 239-54.
4. Hancock, W.S. and J.E. Battersby, *New micro-test for detection of incomplete coupling reactions in solid-phase peptide synthesis using 2, 4, 6-trinitrobenzene-sulphonic acid.* Anal. Biochem., 1976. 71(1): p. 260-264.
